# Incidental Prostate Cancer in Transurethral Resection of the Prostate Specimens in the Modern Era

**DOI:** 10.1155/2014/627290

**Published:** 2014-04-29

**Authors:** Brandon Otto, Christopher Barbieri, Richard Lee, Alexis E. Te, Steven A. Kaplan, Brian Robinson, Bilal Chughtai

**Affiliations:** ^1^Department of Urology, Weill Medical College of Cornell University, New York-Presbyterian Hospital, 425 E 61st Street, New York, NY 10065, USA; ^2^Department of Pathology & Laboratory Medicine, Weill Medical College of Cornell University, New York-Presbyterian Hospital, New York, NY 10065, USA

## Abstract

*Objectives.* To identify rates of incidentally detected prostate cancer in patients undergoing surgical management of benign prostatic hyperplasia (BPH). *Materials and Methods.* A retrospective review was performed on all transurethral resections of the prostate (TURP) regardless of technique from 2006 to 2011 at a single tertiary care institution. 793 men (ages 45–90) were identified by pathology specimen. Those with a known diagnosis of prostate cancer prior to TURP were excluded (*n* = 22) from the analysis. *Results.* 760 patients had benign pathology; eleven (1.4%) patients were found to have prostate cancer. Grade of disease ranged from Gleason 3 + 3 = 6 to Gleason 3 + 4 = 7. Nine patients had cT1a disease and two had cT1b disease. Seven patients were managed by active surveillance with no further events, one patient underwent radiation, and three patients underwent radical prostatectomy. *Conclusions.* Our series demonstrates that 1.4% of patients were found to have prostate cancer, of these 0.5% required treatment. Given the low incidental prostate cancer detection rate, the value of pathologic review of TURP specimens may be limited depending on the patient population.

## 1. Introduction 

Clinical T1 or incidental prostate cancer is defined as clinically inapparent tumor that is neither palpable nor visible by imaging. Clinical T1a and T1b prostate cancer are diagnosed at the time of transurethral resection of the prostate (TURP) for benign prostatic disease. T1a disease involves 5% or less of the resected tissue, whereas T1b disease involves more than 5% of the resected tissue. Prior to the PSA era, up to 27% of prostate cancers were detected incidentally at the time of TURP [1]. With an increase in PSA screening, there has been a decrease in pT1a and pT1b lesions [2].

Along with this shift in incidental prostate cancer distribution with the introduction of PSA, fewer traditional TURPs are being performed as newer techniques, such as laser vaporization, are being adopted [3]. These new technologies do not always provide tissue for pathological examination leading to potentially missed cancers. Some incidental prostate cancers have been shown to be clinically relevant, specifically tumors with a higher Gleason score and stage pT1b [4].

In the context of current screening practices and changing practice patterns, we sought to identify the rates of incidentally detected prostate cancer in TURP specimens.

## 2. Materials and Methods

After obtaining Institutional Review Board (IRB) approval, a retrospective review was performed of all transurethral resections or enucleations of the prostate that provided a tissue specimen between 2006 and 2011. 793 men, aged from 45 to 90 (median age 71), were identified who underwent a transurethral procedure of the prostate that provided a specimen.

All patients were evaluated preoperatively with a digital rectal examination (DRE), PSA screening as indicated by American Urological Association (AUA) guidelines, and prostate biopsy when indicated. Prostate biopsy was performed on all patients with an abnormal PSA or with an abnormal DRE as per surgeon preference. Histopathological results, weight of tissue resected, and amount of tissue were analyzed.

Eight initial cassettes of tissue plus 1 cassette per each additional 10 grams of tissue beyond that were submitted for analysis. In accordance with guidelines set by the College of American Pathologists (CAP), all remaining chips were submitted for evaluation in cases of incidental tumor detection that was Gleason score 6 and involving <5% of tissue [5].

All specimens were analyzed by an uropathologist. Patients with a preoperative diagnosis of prostate cancer were excluded from the analysis (*n* = 22).

## 3. Results

793 men (age 45–90, median 71) were identified by pathology. Twenty-two men were excluded because of a prior diagnosis of prostate cancer. 760 (98.6%) patients had benign prostatic hyperplasia or inflammation on pathology. Eleven (1.4%) patients were found to have prostate cancer on pathology. Ten patients had Gleason grade 3 + 3 = 6 disease and one patient had Gleason grade 3 + 4 = 7 disease. Of these 11 patients, 9 patients had T1a disease and 2 had T1b disease. Seven patients were managed with an active surveillance protocol. One patient underwent external beam radiation. Three patients underwent radical prostatectomy and have no evidence of disease at last followup (Table 1).

The mean weight of tissue resected was 8.1 grams (range 0.5–92.4 grams). The mean percent of tissue submitted for analysis was 96%.

## 4. Discussion

Our study showed an incidental prostate cancer rate of 1.4%. Only two patients had T1b lesions, both of them opting for a surveillance strategy given their age and comorbidities. One patient had Gleason grade 3 + 4 prostate cancer and underwent a radical prostatectomy. This detection rate is lower than several other recently published series; however, it is consistent with the overall decrease in incidental prostate cancer in the PSA era [1].

Prior to the introduction of PSA screening, up to 27% of prostate cancer was detected at the time of TURP [1]. Several studies have compared incidental prostate cancer rates between the pre-PSA and the PSA era. First, Tombal et al. reported a decreased rate of incidental prostate cancer from 27% to 9% when comparing their pre-PSA era to PSA era detection rates in over 1600 patients [1]. They saw a larger decrease in T1b lesions, 15% to 2%, than in T1a lesions, which stayed relatively constant at 3% to 5% [1]. Mai et al. also showed similar results in their review of almost 1000 TURP specimens. They found significant decreases in the overall detection rate, 12.9 to 8%, and the amount of pT1b lesions, 10% to 5% [6]. More recently, Jones et al.'s comparison found a decrease of incidental prostate cancer from 14.9% to 5.2% (pre versus post PSA era) in over 700 patients [7]. They saw significant decreases in both pT1a and pT1b incidental prostate cancer (4.4% to 2.2% and 10.5% to 2.8%, resp.) between the pre-PSA and the PSA eras [7]. These studies indicate that PSA screening has decreased the detection of incidental prostate cancer, specifically T1b lesions. They also suggest that men considering ablative surgical management of BPH are informed that there is a low risk of harboring clinically significant undetected malignancy. Other possible reasons for the reduction in incidental prostate cancer include the decreased rate of surgical management of BPH due to increased use of medical therapy as well as an increased use of ablative therapies, which do not always provide tissue for pathologic analysis in patients who ultimately require surgical management of their BPH [3].

Several studies in addition to ours have looked at the incidental prostate cancer rate in the PSA era. Prior to our findings, detection rates in the PSA era ranged from 4.8% to 16.7% [4, 6–11]. Dellavedova et al. found an incidental prostate cancer detection rate of 7% when they reviewed 100 patients who underwent bipolar TURP [8]. Six patients had Gleason grade 3 + 3 pT1a disease and one patient had Gleason grade 3 + 4 pT1b disease [8]. In Helfand et al.'s study looking at postoperative changes in PSA and PSA velocity in patients undergoing surgical management of BPH, they found an incidental prostate cancer rate of 8.7% in 313 patients who underwent monopolar or bipolar TURP [9]. 20 patients had pT1a disease and 10 had pT1b disease. They also showed that postoperative PSA values decreased less and PSA velocity was higher in patients who had incidental prostate cancer compared to BPH [9]. Voigt et al. found an incidental prostate cancer rate of 11.1% in their study looking to identify risk factors for having clinically relevant prostate cancer discovered incidentally [4]. 3.4% of the patients in their series had clinically relevant prostate cancer, pT1b, or Gleason grade 7–10 disease [4]. Trpkov et al. have reported the highest incidental prostate cancer rate in the PSA era, 16.7%; however, their study included patients with known prostate cancer [10]. A recent multicenter review by Yoo et al. showed an incidental prostate cancer rate of 4.8% in over 1600 patients [11]. They found that in addition to DRE findings, the combination of transitional zone volume and PSA could be useful predictors of incidental prostate cancer [11]. Overall, these studies continue to support both a decreased overall prevalence of incidental prostate cancer and more specifically pT1b lesions in the modern era. In addition, they support the use of technologies that do not provide tissue for pathologic examination at the time of BPH surgical management.

Our detection rate of 1.4% may be lower than the other reported series. One reason may be that since this is the most recent series, a higher proportion of patients may have undergone systematic biopsies as indicated prior to their TURP compared to earlier series which may have included less cores in their biopsy specimens. In addition, the small amount of tissue removed in this study could potentially result in underdetection of prostate cancer. These may help explain why our incidental prostate cancer rate is lower than other published series. Our uropathologist processes and analyzes specimens according to standard procedures.

The natural history of incidental prostate cancer has been studied. Early studies showed that T1a lesions were usually less aggressive than T1b lesions [12]. In their long-term followup of patients with incidental prostate cancer, Tombal et al. showed that T1b lesions are associated with a higher Gleason score and a higher risk of progression [1]. Descazeaud et al. identified five adverse factors associated with progression of T1a tumors; specifically the 5-year progression increased from 12% to 47% if a patient had two or more of the following parameters: pre-op PSA ≥ 10, post-op PSA ≥ 2, prostate weight ≥ 60 g, weight of resected tissue ≥ 40 g, and Gleason score ≥ 6 [13].

Often following incidental diagnosis of prostate cancer after TURP, patients may undergo additional diagnostic procedures to provide further assessment of the cancer. In a study by Lee et al., 63 patients underwent TRUSBx or radical prostatectomy procedures after being diagnosed with incidental prostate cancer. Of the 22 patients who underwent TRUSBx, 54% were downgraded and most of these were benign. Lee et al. found that in most cases, TRUSBx did not provide enough additional information to be warranted for many patients pursuing treatment for TURP-diagnosed incidental prostate cancer [14].

There is some conflicting data on outcomes following radical prostatectomy for incidental prostate cancer according to T1a versus T1b stage. Capitanio et al. showed that PSA before and after surgery for BPH and Gleason score were predictors of residual cancer at the time of radical prostatectomy and of biochemical recurrence. Interestingly, T1a and T1b stage were not predictive [15]. Magheli et al. also showed that T1 subclassification did not predict BCR [16]. In contrast, Helfand et al. found that while overall biochemical free recurrence, overall survival, and cancer specific survival were excellent for patients with incidental prostate cancer, patients with T1b had a marginal but significant decrease in 10-year disease specific survival [17]. Further studies and longer followup are needed to sort out the significance of T1a versus T1b staging.

Taking the natural history and outcomes data available on incidental prostate cancer, the European Association of Urology (EAU) has provided specific guidelines for the management of incidental prostate cancer. The EAU recommends active surveillance or watchful waiting for patients with T1a tumors and patients with T1b tumors if Gleason score is 6 or less and the life expectancy of the patient is less than 10 years. For patients with T1b tumors and a life expectancy of more than 10 years, radical prostatectomy is recommended. The AUA guidelines do not specifically address the management of T1a or T1b lesions. For low risk prostate cancer, they propose that active surveillance, brachytherapy, external beam radiotherapy, and radical prostatectomy are appropriate therapy options. The patients in our study are being managed in accordance with the EAU guidelines. The two patients with T1b lesions opted for surveillance given that their life expectancy was less than 10 years. One patient with Gleason 3 + 4 disease underwent a radical prostatectomy and is currently NED. Of the remaining eight patients with T1a, Gleason grade 3 + 3 = 6 lesions, five chose surveillance, one chose radiation, and two chose radical prostatectomy.

This study has several limitations. First, it was a retrospective study that included many different surgical treatment modalities, potentially modifying the amount of tissue that was submitted for review. Secondly, it was a heavily prescreened population that may not be generalizable to community setting.

## 5. Conclusions

We demonstrated an incidental prostate cancer rate of 1.4% in the PSA era. Given the low incidental prostate cancer detection rate, the value of pathologic review of TURP specimens may be limited depending on the patient population.

## Figures and Tables

**Table 1:** Incidental prostate cancer patient characteristics.

Patient	Age	Gleason grade	Stage	Management	Comments
1	72	3 + 3 = 6	T1a	AS	
2	85	3 + 3 = 6	T1b	AS	
3	57	3 + 3 = 6	T1a	AS	Repeat bx negative
4	66	3 + 3 = 6	T1a	AS	
5	68	3 + 3 = 6	T1a	RP	3 + 3 at RP; NED
6	80	3 + 3 = 6	T1b	AS	
7	53	3 + 3 = 6	T1a	AS	Repeat bx negative
8	78	3 + 3 = 6	T1a	AS	
9	70	3 + 3 = 6	T1a	AS→XRT	
10	59	3 + 3 = 6	T1a	RP	3 + 3 at RP; NED
11	67	3 + 4 = 7	T1a	RP	

AS: active surveillance; RP: radical prostatectomy; XRT: external beam radiation.
